# Retrograde signalling in a virescent mutant triggers an anterograde delay of chloroplast biogenesis that requires GUN1 and is essential for survival

**DOI:** 10.1098/rstb.2019.0400

**Published:** 2020-05-04

**Authors:** Naresh Loudya, Tolulope Okunola, Jia He, Paul Jarvis, Enrique López-Juez

**Affiliations:** 1 Department of Biological Sciences, Royal Holloway University of London, Egham TW20 0EX, UK; 2 Department of Plant Sciences, University of Oxford, South Parks Road, Oxford OX1 3RB, UK

**Keywords:** chloroplast, retrograde signalling, CUE8, GUN1, RNA editing, nucleoid

## Abstract

Defects in chloroplast development are ‘retrograde-signalled’ to the nucleus, reducing synthesis of photosynthetic or related proteins. The *Arabidopsis* *cue8* mutant manifests virescence, a slow-greening phenotype, and is defective at an early stage in plastid development. Greening cotyledons or early leaf cells of *cue8* exhibit immature chloroplasts which fail to fill the available cellular space. Such chloroplasts show reduced expression of genes of photosynthetic function, dependent on the plastid-encoded polymerase (PEP), while the expression of genes of housekeeping function driven by the nucleus-encoded polymerase (NEP) is elevated, a phenotype shared with mutants in plastid genetic functions. We attribute this phenotype to reduced expression of specific PEP-controlling sigma factors, elevated expression of *RPOT* (NEP) genes and maintained replication of plastid genomes (resulting in densely coalesced nucleoids in the mutant), i.e. it is due to an anterograde nucleus-to-chloroplast correction, analogous to retention of a juvenile plastid state. Mutants in plastid protein import components, particularly those involved in housekeeping protein import, also show this ‘retro-anterograde’ correction. Loss of CUE8 also causes changes in mRNA editing. The overall response has strong fitness value: loss of GUN1, an integrator of retrograde signalling, abolishes elements of it (albeit not others, including editing changes), causing bleaching and eventual seedling lethality upon *cue8 gun1*. This highlights the adaptive significance of virescence and retrograde signalling.

This article is part of the theme issue ‘Retrograde signalling from endosymbiotic organelles’.

## 1. Introduction

Four decades ago plastid-to-nucleus communication was first observed as a reduction in synthesis rate of a nuclear-encoded chloroplast protein in a chloroplast ribosomes-deficient mutant [1]. The phenomenon triggering the reduction was presumed to be of fitness value on the basis of cellular economy, preventing the wasteful process of synthesizing proteins when their cellular organelle target was unprepared to receive them. The process has been named ‘retrograde signalling’ given that the majority of proteins needed for organelle biogenesis are encoded in the nucleus, which therefore exerts the initial ‘anterograde control’ [2,3]. Genetic or chemically induced defects in pigment biosynthesis, chloroplast genetic function or chloroplast protein import all result in a ‘biogenic retrograde communication’ response [3,4]. The extent of responses has been documented at the global nuclear gene expression level [5,6]. It is distinct from the responses caused by later, ‘operational retrograde signals', which occur when functional chloroplasts operate under stress [4].

Virescent mutants are a class of plastid-defective mutants which exhibit a slow-greening phenotype, in which young cells in leaf primordia and at the proximal region of developing leaves show reduced chlorophyll accumulation compared with more mature, distal cells. The few virescent mutants for which the molecular basis has been identified are defective in plastid protein homeostasis [7,8] or are auxotrophs for plastid-based nucleotide metabolism [9]. Plastids being central hubs of metabolism in plants and hosts to a very large proportion of cellular protein explains the fact that many metabolic defects will manifest first as plastid biogenesis deficiencies. A genetic effort to identify components involved in light signalling on the basis of its control of chloroplast development [10] led to the identification of a number of *cab-underexpressed* (*cue*) mutants, several among which exhibited a virescent phenotype [11]. In one of those mutants, the ultrastructure of plastids showed a transition from proplastid to fully differentiated chloroplast which is difficult to capture in wild-type (WT) plants. Among them, the *cue8* mutant showed a very strong defect in both dark-grown etioplasts and light-grown chloroplasts [11,12]. The phenotype of reduced expression of photosynthesis-associated nuclear genes (PhANGs) of these mutants was due to interorganellar communication resulting from the impaired plastid development [11,12].

Chloroplast-to-nucleus retrograde signalling has remained a captivating subject since its discovery. The *Genomes Uncoupled 1* gene (*GUN1*) [5] was identified through an elegant genetic screen, encodes a pentatricopeptide domain-containing important chloroplast protein which associates with chloroplast ribosomes and envelope protein import complexes, and has turned out to be a central integrator of signals triggering this interorganellar communication [13]. The molecular function of GUN1 as a likely sensor of protein homeostasis [14,15] and a regulator of chloroplast mRNA editing [16,17] is beginning to be uncovered.

In spite of this progress, the very biological role of biogenic retrograde communication is less well understood than that of operational signals. In this study, we aimed at understanding the nature of the virescence phenotype and its impact on chloroplast development. In the process, we observed a remarkable chloroplast-nucleus-chloroplast response which we term ‘retro-anterograde’ communication, aspects of which had previously been observed but others are described for the first time; became struck by the parallels between this response and the early stages of undisturbed chloroplast development; observed aspects of the response which depend on GUN1 and others which do not; and uncovered evidence for a fitness value of retrograde biogenic signalling, which places it in a new organelle developmental light.

## 2. Material and methods

### (a) Plant material and growth conditions

The *Arabidopsis* *cue8* mutant [11,12] and its WT pOCA108, in the Bensheim ecotype, have been previously described. *ppi1*, *toc132 toc120* and *gun1*-1 mutants, in the Col-0 background, have also been described [18–20]. The *cue8* mutation was introgressed by backcrossing six times into Col-0, and the resulting line, *cue8*^Col^, used to generate the *cue8 gun1* double mutant. Mutations were genotyped by polymerase chain reaction (PCR), followed or not by digestion, as described in electronic supplementary material, table S1. Plants were grown on soil under photoperiod conditions and seedlings *in vitro* on MS media supplemented with 1% sucrose under continuous white light (100 µmol m^−2^ s^−1^) as described in Vinti *et al.* [12].

### (b) Analysis of plastid development

Observation of the proportion of each cell occupied by chloroplasts used plant material subjected to fixation (3.5% v/v glutaraldehyde) and cell separation in 0.1 M EDTA (65°C, 1 h), followed by examination using Nomarski microscopy (Nikon Optiphot-2, Plan Apo ×20 and Plan Fluor ×40 objectives, Micropublisher 5.0 camera). Cells were always selected towards the distal half of cotyledons or leaves. NIS-Elements AR 2.30 software (Nikon) was used for live measurements of the cell and chloroplast plan area, and organelle count. Cell area, mean chloroplast area, total chloroplast area (total chloroplast number × mean chloroplast area) and chloroplast compartment or cell index (total chloroplast area/cell area) was obtained from triplicate biological samples (separate plants) of each genotype. Cellular and chloroplast area was measured by selecting 10 random objective-facing chloroplasts per cell and 3–5 mesophyll cells per replicate. Total chloroplast counts were obtained by live counting on different planes, completely moving out of focus and slowly focusing inwards until losing the focus again, marking only clearly visible plastids in different planes [21]. To visualize nucleoids, the fixed cells were mounted directly in DNA-binding 4′,6-diamidino-2-phenylindole (DAPI, Partec cystain UV precise P Solution 2, Sysmex) and subjected to fluorescence microscopy (Nikon H600 L Ni-E, x60 Plan Apo oil immersion objective), with UV excitation and blue emission filters.

### (c) Gene expression analysis, RNA editing and plastid genome copy number

Gene expression was quantified in a two-step reverse transcriptase-quantitative polymerase chain reaction (RT-qPCR). The reverse-transcribed cDNA (QuantiTect® Reverse Transcription kit, using oligo dT and random primers, Qiagen) was analysed through qPCR using the SyGreen Mix Lo-ROX (PCR Biosystems) in the Rotorgene Q real-time PCR cycler (Qiagen). Primers were as described in electronic supplementary material, tables S2 and S3. The transcript level of selected genes was normalized to the levels of a reference gene (*UBQ10*). Each mutant was compared with the respective WT and calculated individually (E^−Cttest^/E^−Ctcontrol^, where E = efficiency and Ct = take-off value as calculated by RotorgeneQ).

Chloroplast genome copy numbers were determined by qPCR using the standard curve analysis and primers as described in electronic supplementary material, table S4. Standards of known concentration (25 pg µl^−1^ to 0.0025 pg µl^−1^) were prepared from purified (QIAquick kit, Qiagen) PCR-amplified products representing two nuclear genes (*HO1*, *CHS*) and three chloroplast genes (*rbcL*––large single-copy region, *ndhG*––small single-copy region and *ycf2*––inverted repeat region). Total DNA extracted from plant tissue was 10-fold diluted for nuclear genes and 100-fold for the quantitation of plastid genes. Samples were subjected to qPCR analysis along with their standards of known concentration. The ratio of plastid DNA/genomic DNA quantified with mean values of three chloroplast genes and two nuclear single-copy genes ((*rbcL* + *ndhG* + *ycf2/2*)/3)/((*HO1* + *CHS*)/2) resulted in absolute quantities of chloroplast DNA copies per haploid genome.

Editing of chloroplast RNA was analysed and calculated according to a published protocol [17] with modifications, by sequencing the purified (QIAquick, Qiagen) PCR-amplified products generated using high-fidelity DNA Polymerase (Phusion, Thermo Scientific) and modified PCR conditions (98°C for 3 min, (98°C for 30 s, 52°C for 45 s, 72°C for 1 min) × 33 cycles and 72°C for 5 min) from three independent cDNA templates. Primers used for PCR amplification and sequencing in the RNA editing analysis are listed in electronic supplementary material, table S5.

### (d) Nuclear DNA ploidy analysis

Average nuclear DNA content per cell of the developing seedlings was measured using flow cytometry (Sysmex CyFlow® Space, Sysmex, UK), broadly according to Mohammed *et al.* [22], by counting a minimum of 16 000 nuclei per biological replicate (6–7 replicates per time point).

## 3. Results

### (a) *Cue8* is a strong virescent mutant, exhibiting delayed greening

**Figure 1.**
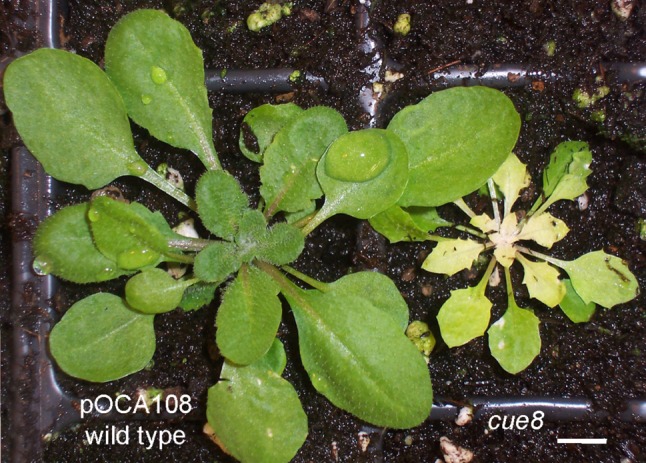
The *Arabidopsis* *cue8* mutant exhibits a virescent, slow-greening phenotype. Plants of the pOCA108 line in the Bensheim ecotype (parental, WT) and the *cue8* mutant were grown on soil for four weeks under 16 h photoperiods and 180 µmol m^−2^ s^−1^ white light, before being photographed. (Online version in colour.)

**Figure 2.**
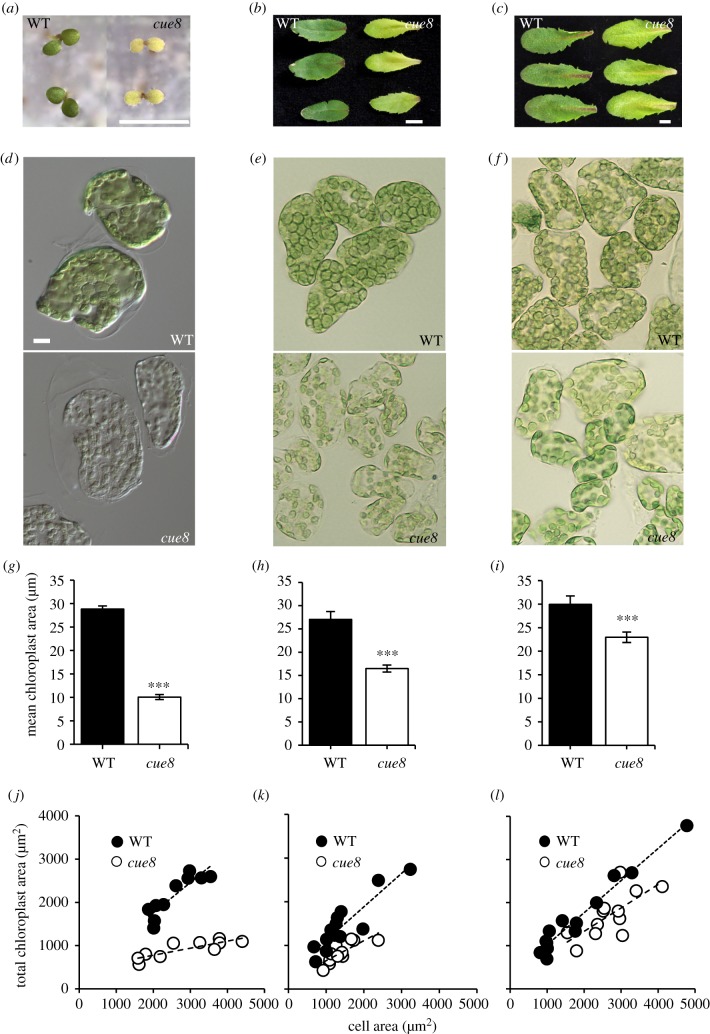
*cue8* cells have a reduced, gradually building total cellular chloroplast compartment. (*a*) Images of developmentally matched seedlings, 5-day-old WT and 6-day-old *cue8*, grown on sucrose-containing media under continuous light. (*b*) Young leaves of WT and *cue8* soil-grown plants, as in figure 1. (*c*) Fully mature leaves of WT and *cue8* soil-grown plants. Scale bar (for *a*,*b* and *c*), 5 mm. (*d*) Individual cells of WT and *cue8*, isolated and separated from cotyledons of seedlings as in (*a*). (*e*) Individual cells of WT and *cue8* from young leaves (distal half) as in (*b*). (*f*) Individual cells of WT and *cue8* mature leaves as in (*c*). Scale bar, 10 µm (all microscopy images at same scale). (*g*,*h*,*i*) Mean plan area of individual chloroplasts, measured in cells represented by those in (*d*,*e*,*f*), respectively. The area of 10 chloroplasts per cell, from 10 to 13 cells, obtained from three separate cotyledons or leaves, was quantified under live imaging. The bars represent average and the error bars s.e.m. between cells. *** indicates the result of individual *t*-tests between *cue8* and WT (*p* < 0.001). (*j*,*k*,*l*) Scatter plots representing the total chloroplast plan area in a cell (computed as the total number of chloroplasts × average plan area of those chloroplasts) as a function of the plan area of that cell, measured in cells represented by those in (*d*,*e*,*f*). Linear regression lines are shown for WT and *cue8*. The slope of those lines was used to estimate the chloroplast compartment or ‘cell index’ (see text). (Online version in colour.)

The *cue8* mutant manifests a very early defect in chloroplasts' development, as seen both before and after de-etiolation [11,12], and a very pronounced defect in the expression of PhANGs, but not other light-dependent genes [12,23]. We focused our attention on the mutant in an attempt to understand the nature of the virescent phenotype (the exact identity of the *CUE8* gene will be the subject of a separate report). Indeed, rosettes of *cue8* are characterized by very young leaves which range from pale to almost albino, yet greening occurs gradually, resulting in a gradient from older (tip) to younger (base) cells as leaves develop (figure 1). Young seedlings of *cue8* are also almost albino (figure 2; electronic supplementary material, figure S2).

### (b) Immature chloroplasts of *cue8* fail to fill the available cellular space, but the cell's chloroplast compartment builds gradually

The virescent phenotype of *cue8* could be explained by the delayed development of individual chloroplasts, by a failure of chloroplasts to fill the available cell's cytoplasm, or both [21]. We examined by quantitative Nomarski microscopy the cellular population of chloroplasts (figure 2*a,d,g,j*) of cotyledon mesophyll cells of developmentally matched seedlings of WT (5 days) and *cue8* (6 days, figure 2*a*). WT chloroplasts had an average individual plan area of 29 µm^2^ (figure 2*g*) and essentially filled the available cellular space (figure 2*d,j*). By contrast, cotyledon mesophyll cells of very pale *cue8* seedlings contained early-developing chloroplasts (which could be described as proplastids or, more specifically, eoplasts) about one third in size, in similar or even slightly elevated numbers to those of the WT (electronic supplementary material, figure S1) and which in total occupied about 40% of the cell plan area (figure 2*d*,*g*,*j*). Note that while we measured cell and chloroplast plan areas, rather than volumes, the fact that cells are filled by a large central vacuole, surrounded by a layer of chloroplast-containing cytoplasm, means that the organelle share can fairly be treated as a share of a two-dimensional space.

Later stages of the virescent phenotype were monitored by examining mesophyll cells of relatively young and nearly mature leaves of *cue8* (figure 2*b,c,e,f,h,i*). Indeed, in mesophyll cells of young leaves *cue8* chloroplasts had grown to about 60% of the individual size and of the total cellular occupancy of those of the WT while, in the adult leaves of *cue8*, chloroplasts reached over 75% of the size of WT and occupied 70% of the cell plan area (again, the number of chloroplasts per cell was invariant or slightly elevated, electronic supplementary material, figure S1). Hence the *cue8* chloroplast population never reaches a cellular occupancy identical to that in the WT, but it builds gradually and reaches approaching values.

### (c) Compensatory pattern of transcript accumulation of *cue8* chloroplasts and its nuclear basis

**Figure 3.**
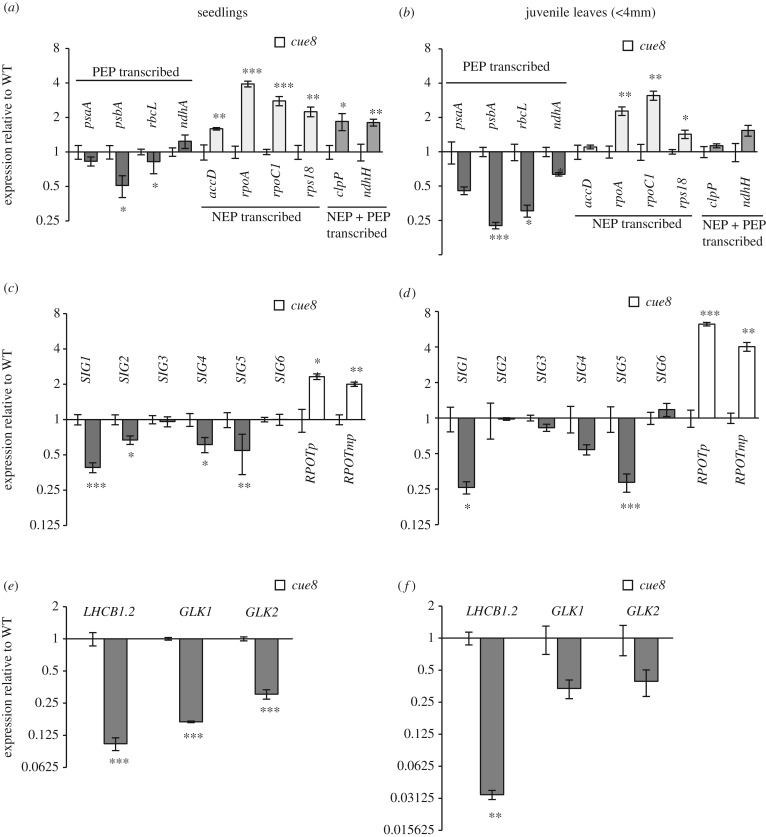
Molecular genetic phenotype of *cue8* cells and their chloroplasts, which exhibit a ‘compensatory’ gene expression response. (*a*) Levels of plastid genome-encoded transcripts in 5-day-old WT and 6-day-old *cue8* seedlings, quantified by qRT-PCR and normalized to the levels of a constitutive transcript (see methods), expressed on a log_2_ scale relative to the levels of the same plastid-encoded transcript in WT (level in WT given a value of 1). The transcripts are grouped according to the plastid RNA polymerase (plastid-encoded polymerase, PEP; nuclear-encoded polymerase, NEP) primarily responsible for their expression. Averages of three biological samples, each measured in two technical replicates. Error bars represent s.e.m. between biological replicates. The WT always has an expression relative value of 1, hence no bar appears, but the s.e.m. between WT samples is also given. Asterisks represent the level of significance of the difference in *t*-tests between *cue8* and WT (*p < 0.05, **p < 0.01, ***p < 0.001). (*b*) Levels of plastid genome-encoded transcripts in juvenile (less than 4 mm in length) leaves of WT and *cue8*, quantified and expressed as in (*a*). (*c*) Transcript levels in WT and *cue8* seedlings of nuclear-encoded genes for PEP specificity-determining sigma factors (*SIG*) and for NEPs (*RPOTp* and *RPOTmp*), expressed as in (*a*). (*d*) Transcript levels in juvenile leaves of WT and *cue8* of nuclear-encoded *SIG* and *RPOT* genes, expressed as in (*b*). (*e*) Transcript levels in WT and *cue8* seedlings of nuclear-encoded genes for a prototypical photosynthesis-associated nuclear gene, *LHCB1.2*, and for the *LHCB1.2*-driving GLK transcription factors, expressed as in (*a*). (*f*) Transcript levels in juvenile leaves of WT and *cue8* of nuclear-encoded *LHCB1.2* and GLK transcription factors, expressed as in (*b*).

The virescent phenotype of *cue8* involves the reduced expression of nuclear genes specifically associated with photosynthesis [12,23]. Many of those genes encode products which assemble as photosynthetic complexes together with the products of chloroplast-encoded genes. To understand the nature of virescence, and given the underdeveloped chloroplasts, we monitored the expression of representative genes encoded in the plastid genome. In initial experiments, we did this comparing 5-day-old seedlings of both *cue8* and the WT (electronic supplementary material, figure S2). Different genes showed strikingly different responses, in a pattern which reflected the RNA polymerase primarily responsible for their expression: transcript levels of several genes primarily transcribed by the plastid-encoded polymerase (PEP) were distinctly reduced, while those for genes transcribed by the nucleus-encoded, T7 or mitochondrial-type polymerase (NEP) were elevated (electronic supplementary material, figure S2). Because *cue8* seedlings develop somewhat slower than WT, we repeated those experiments with 6-day-old mutant and 5-day-old WT seedlings. In this case, the reduction in PEP-dependent transcript levels was attenuated, while the elevation of NEP- and ‘NEP- or PEP’- dependent transcripts remained (figure 3*a*). Samples of very young, ‘juvenile’ leaves, less than 4 mm long, of mutant (almost albino) and WT (green) were also examined and manifested essentially the same response (figure 3*b*). This contrast in expression of plastid-encoded genes transcribed by both polymerases was first observed when an essential PEP subunit gene, *rpoB*, was knocked-out in the tobacco plastid genome [24]. The expression of both groups of chloroplast-encoded genes ultimately depends on nuclear-encoded factors, since even for the PEP, the promoter-selecting sigma factors are nucleus encoded. The contrasting expression differences of plastid-encoded genes could be explained by opposite expression changes of the NEP genes and of the PEP-controlling sigma factor genes. Indeed we observed the reduced expression of *SIG1* and *SIG5*, and elevated the expression of the two *RPOT* genes for chloroplast NEP, in both seedlings and juvenile leaves (figure 3*c*,*d*). This accompanied in both cases the expected, virescence-associated reduction in *LHCB1.2*, a prototypical PhANG, itself dependent on PhANG-transcribing GLK transcription factors (figure 3*e*,*f*).

Given that NEP-dependent genes primarily encode proteins of the plastid genetic machinery (including *rpoA* and *rpoC1*, encoding subunits of the PEP itself), the elevated levels of NEP-dependent transcripts have been considered a ‘compensatory’ mechanism to attempt to correct a detected plastid protein synthesis defect [24–26]. Our observations show that this change is actually the result of an alteration of nuclear gene expression.

### (d) The underdeveloped chloroplasts of *cue8* accumulate normal numbers of copies of the plastid genome

**Figure 4.**
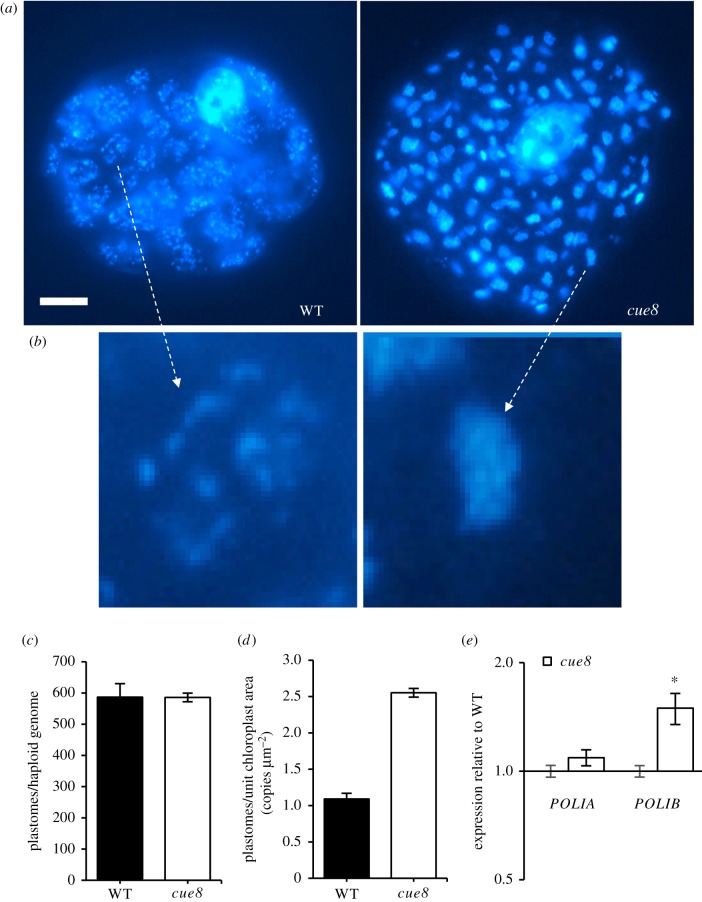
Defective *cue8* chloroplasts have densely packed nucleoids and normal total number of plastome copies per cell. (*a*) Representative cells of WT and *cue8* stained for double-stranded DNA with DAPI and observed under fluorescence microscopy, to visualize nuclear DNA and the nucleoids in plastids. Scale bar, 10 µm. (*b*) Magnification of individual plastids in (*a*), as indicated by the arrows. (*c*) Number of copies of the plastid genome in WT and *cue8*, expressed per copy of the haploid nuclear genome. (*d*) Number of copies of the plastid genome per unit chloroplast plan area (calculated assuming four copies of the haploid genome per cell; see text). Error bars represent s.e.m. (*e*) Transcript levels of the two organellar DNA polymerases in seedlings of WT and *cue8*, quantified and expressed as in figure 3*a*. (Online version in colour.)

It is nevertheless surprising that underdeveloped chloroplasts of *cue8* manage to accumulate levels of NEP-dependent transcripts two- to fourfold greater than those of the WT. The NEP operates on plastid DNA (cpDNA), present in membrane-associated nucleoprotein assemblies called nucleoids, as a template. We reasoned that underdeveloped chloroplasts of *cue8* cotyledon cells, occupying a third of the cellular space they would do in the WT, would have reduced levels of cellular cpDNA. To our initial surprise, this was not the case (figure 4): cpDNA levels in *cue8* seedlings, measured by qPCR relative to the haploid nuclear genome, were identical to those in WT (figure 4*c*). Our assay quantified targets in the plastid genome encoded in both single copies and the inverted repeat regions, ruling out cpDNA rearrangements producing artefactual data. Given that in seedlings the plastids were smaller, this also meant that the number of copies of the chloroplast genome expressed per unit plan area of chloroplasts in the cell (calculated assuming an average of four haploid genomes per cotyledon cell [27]) was also substantially higher in the mutant (figure 4*d*). Expressed in a different manner, the average chloroplast in cotyledon cells carried a similar number of copies of the cpDNA in WT and *cue8*, 31 and 26, respectively (*cue8* cells contain a slightly elevated number of chloroplasts). Indeed, DAPI fluorescence-staining of DNA showed that mutant plastids were packed with nucleoids, which appeared much more dispersed in WT chloroplasts (figure 4*a*,*b*).

Given the parallels between the expression of NEP- or PEP-encoded genes in *cue8* and that at early stages of undisturbed chloroplast development, we asked whether the elevated content of cpDNA in *cue8* chloroplasts in spite of their undeveloped state was in fact simply a common feature of developing chloroplasts. To carefully address this, we quantified cpDNA in developing WT seedlings, beginning from stages shortly after germination, in which cotyledons are barely emerging and greening (electronic supplementary material, figure S3a–d). We found that the numbers of copies of cpDNA per haploid genome were strikingly constant (electronic supplementary material, figure S3e), from very early stages, demonstrating that the accumulation of the chloroplast genome is a very early event in chloroplast development. However, as seedlings developed, and due to the expansion of cotyledon cells and concomitant endoreduplication (electronic supplementary material, figure S3*a*–*d*,*f*), the average ploidy per nucleus (and therefore per cell) for whole seedlings increased up to day 5, and therefore some cpDNA replication, approximately an additional third, can be concluded to have also taken place beyond that occurring in very early plastids (electronic supplementary material, figure S3 g). The fact that *cue8* cells, of WT-like size but with still underdeveloped chloroplasts, had already achieved the levels of cpDNA equivalent to those of the WT does indicate that additional DNA synthesis occurs in the mutant organelles.

The maintenance of cpDNA levels in the mutant, in spite of the reduced plastid development (which we interpret to be a combination of ‘juvenility’ and additional cpDNA replication), was therefore a further aspect of the chloroplast gene expression ‘compensatory mechanism’ observed in *cue8*. It correlated with mildly elevated expression levels of the *POL1B* gene encoding organellar DNA polymerase, although this difference was also small (figure 4*e*).

### (e) Early chloroplast impairments by loss of housekeeping proteins more consistently trigger the compensatory gene expression phenotype

**Figure 5.**
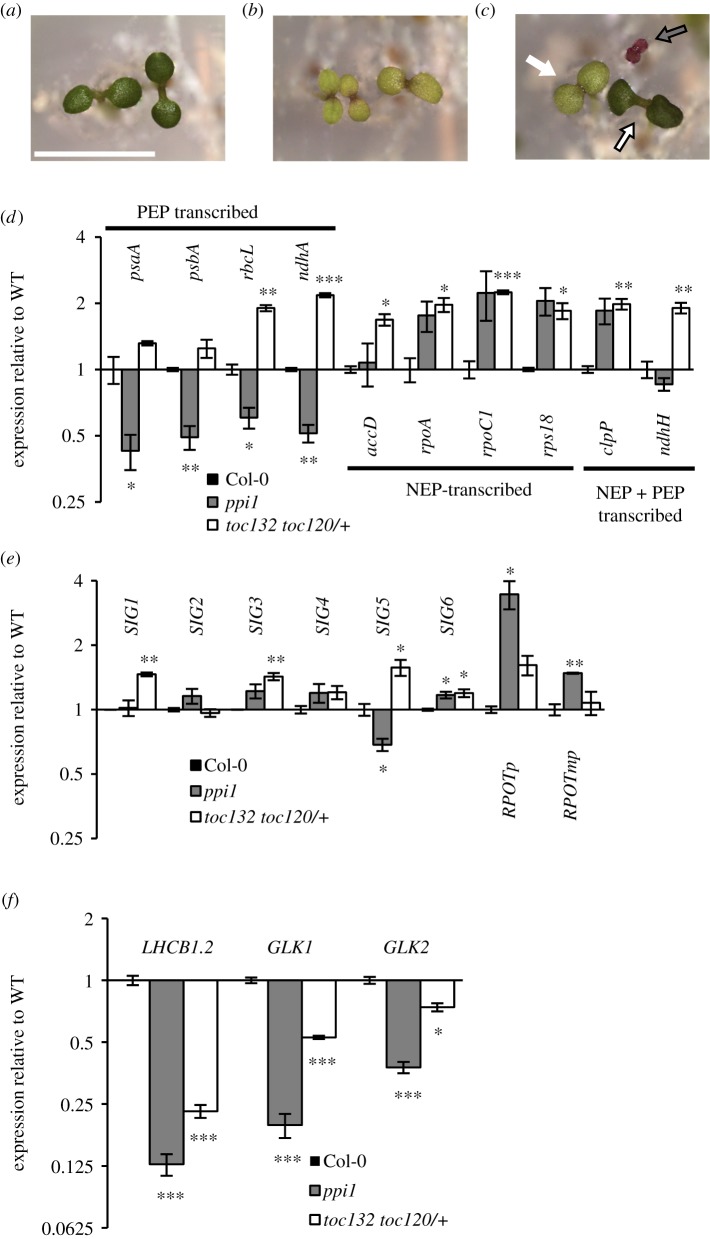
Mutants impaired primarily in photosynthesis or housekeeping chloroplast functions, as a result of selective protein import defects, share the *cue8* compensatory gene expression phenotype, although the loss of housekeeping chloroplast function does so more consistently. (*a–c*) Five-day-old seedlings of Col-0 WT (*a*), *ppi1* (*b*) and segregating seedlings from a *toc132 −/− toc120 −/+* parent (*c*); scale bar, 5 mm. In (*c*), white, black-edged arrow: *toc132 −/− toc120 +/+*; white arrow: *toc132 −/− toc120 −/+*; grey arrow: *toc132 −/− toc120 −/−*. Only seedlings represented by the white arrow were harvested. (*d*) Levels of plastid genome-encoded transcripts in 5-day-old seedlings of Col-0 WT and mutant genotypes, quantified by qRT-PCR, normalized and displayed as in figure 3*a*. (*e*) Transcript levels in WT and mutant seedlings of nuclear-encoded genes for PEP specificity-determining sigma factors (*SIG*) and for NEPs (*RPOTp* and *RPOTmp*), expressed and displayed as in figure 3*c*. (*f*) Transcript levels in WT and mutant seedlings of nuclear-encoded genes for a prototypical photosynthesis-associated nuclear gene, *LHCB1.2*, and for the *LHCB1.2*-driving GLK transcription factors, expressed and displayed as in figure 3*e*. (Online version in colour.)

We asked what kind of plastid defect was responsible for triggering this compensatory response. In an attempt to achieve contrasting chloroplast defects, we took advantage of the fact that protein import into developing chloroplasts has been shown to use two types of outer envelope translocon, with differing subunit composition, and resulting in contrasting phenotypes of the loss of function [2,19]: the *ppi1* mutant, defective in *TOC33*, is impaired preferentially in the import of photosynthetic proteins; by contrast, plants deprived of *TOC132* and heterozygous for the loss of *TOC120* (identified from a segregating population) are preferentially defective in the import of housekeeping proteins (figure 5*a–c*). We examined both young seedlings (figure 5*d*–*f*) and very early leaves (less than 4 mm in length) of these mutants (electronic supplementary material, figure S4), which exhibited somewhat comparable greening defects. Relative to WT, *ppi1* showed a reduction in the expression of PEP-transcribed genes in seedlings and very young leaves, while an elevation of NEP-driven transcripts was observed only in seedlings. By contrast, *toc132 toc120*/*+* showed a consistent elevation of NEP-driven transcripts in seedlings and very young leaves. For PEP-driven transcripts, there was no change (young leaves) or an actual elevation (seedlings) in that genotype (figure 5*d*; electronic supplementary material, figure S4). Expression levels of the PEP-controlling sigma factors or the NEP genes themselves broadly followed these patterns (figure 5*e*; electronic supplementary material, figure S4). Overall, it appears both genotypes can exhibit the ‘compensatory’ elevation of NEP and NEP-driven expression, although to different extents at different stages, and loss of primarily ‘housekeeping’ proteins in chloroplasts triggers it more consistently. Accordingly, the loss of *ppi1* had a greater impact on the loss of the representative PhANG and of its corresponding GLK drivers (figure 5*f*).

**Figure 6.**
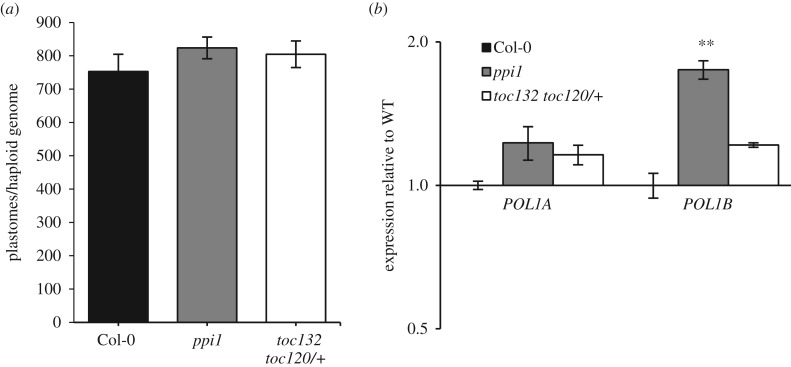
Mutants impaired primarily in photosynthesis or housekeeping chloroplast functions maintain the number of copies per cell of the plastid genome. (*a*) Number of copies of the plastid genome in WT and the mutants indicated, expressed per copy of the haploid nuclear genome. (*b*) Transcript levels of the two organellar DNA polymerases in seedlings of each genotype, quantified and expressed as in figure 3*a*.

The elevated NEP-driven transcription in seedlings was, once again, made possible in these plastid-impaired genotypes by the maintenance of number of cpDNA copies (figure 6*a*), something for which a mild elevation of expression of *POL1B* may have contributed to in *ppi1* (figure 6*b*).

### (f) Survival of *cue8* seedlings necessitates GUN1 activity, responsible for elements of the retro-anterograde control

Corrective action (delay) from nuclear activities following the chloroplast defect necessitates an initial relaying of information to the nucleus, i.e. the action of retrograde chloroplast-to-nucleus signals. Our current understanding of ‘biogenic’ retrograde signals derives almost entirely from the isolation of loss-of-function *gun2*-*gun5* mutants and the gain of function *gun6^D^* mutant, all of them involved in tetrapyrrole metabolism, and of the *gun1* mutant; only the *gun1* mutant exhibits partial uncoupling of nuclear gene expression when chloroplasts are defective by the loss of activity of the genetic machinery (by lincomycin, a chloroplast translation inhibitor) [3,28]. An obvious question is whether GUN1 is involved in the ‘corrective’ response accompanying the virescence in *cue8*.

**Figure 7.**
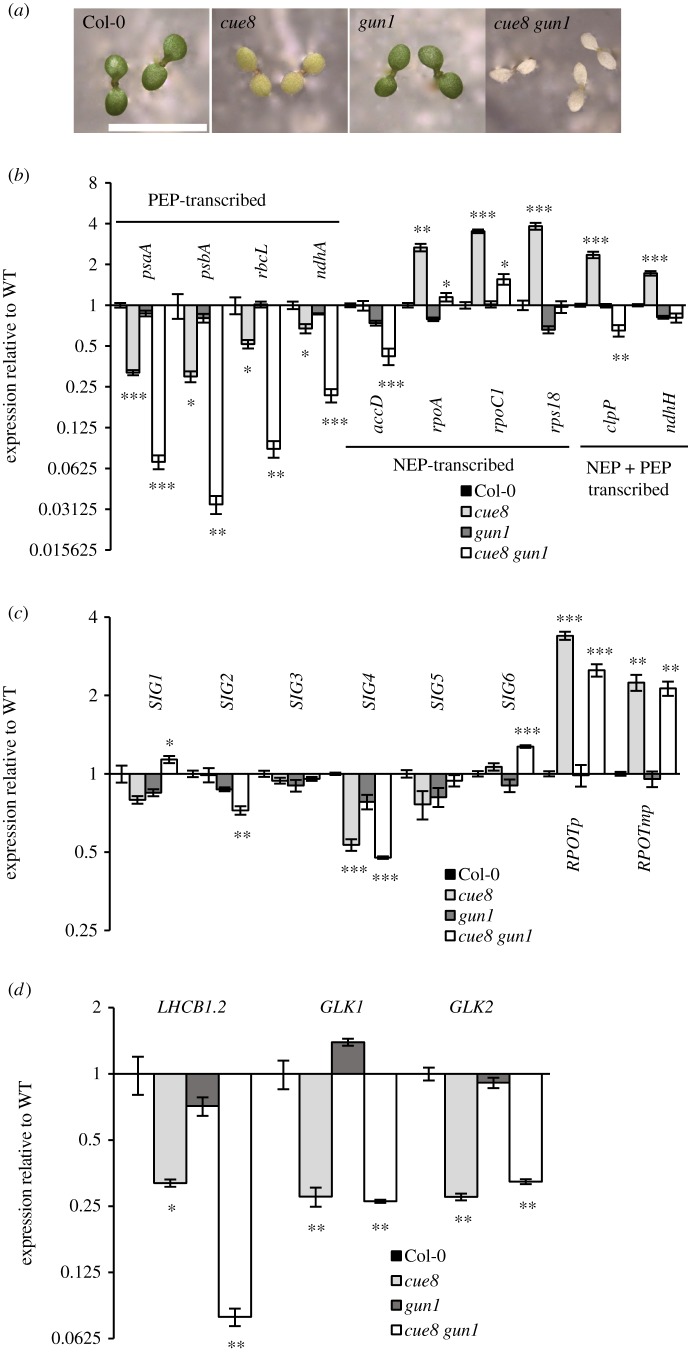
Survival of *cue8* seedlings necessitates GUN1 activity, which is required for some elements of the ‘compensatory’ plastid gene expression response but not others. (*a*) Five-day-old seedlings of WT and *gun1*, 6-day-old seedlings of *cue8^Col^* and 7-day-old seedlings of *cue8 gun1*. Scale bar, 5 mm. (*b*) Levels of plastid genome-encoded transcripts in 5-day-old seedlings of Col-0 WT and mutant genotypes, quantified by qRT-PCR, normalized and displayed as in figure 3*a*. (*c*) Transcript levels in WT and mutant seedlings of nuclear-encoded genes for PEP specificity-determining sigma factors (*SIG*) and for NEPs (*RPOTp* and *RPOTmp*), expressed and displayed as in figure 3*c*. (*d*) Transcript levels in WT and mutant seedlings of nuclear-encoded genes for a prototypical photosynthesis-associated nuclear gene, *LHCB1.2*, and for the *LHCB1.2*-driving GLK transcription factors, expressed and displayed as in figure 3*e*. Note that while the reduction of *LHCB1.2* expression in the double mutant is severe, and the elevated expression of nuclear-encoded NEPs is also very clear, levels of transcripts of chloroplast-encoded, NEP-transcribed genes are not increased. (Online version in colour.)

We reasoned this question would be answered by generating *cue8 gun1* double mutants and asking whether the ‘compensatory’ response remained present. To our initial surprise, progenies of *gun1 cue8*/*+* plants yielded one quarter of albino, very weak seedlings among *gun1*-looking ones. Progenies of *cue8 gun1*/*+* seedlings also generated a quarter fully albino seedlings among *cue8* ones. Genotyping confirmed the albinos to be double mutants (figure 7*a*).

While the double mutant seedlings were very weak, we decided to examine them for evidence of the ‘compensatory’ response. Monitoring of chloroplast gene expression revealed a very strong decrease in the levels of photosynthetic, PEP-driven transcripts in the double mutant, much greater than that in *cue8*. By contrast, the elevation of the NEP-driven transcripts, strong in *cue8*, was almost completely absent in the double (figure 7*b*). Some response was found in the transcript levels of sigma factors, but the elevated levels of transcripts of the NEPs themselves were as visible in *cue8 gun1* as they were in *cue8* (figure 7*c*). *LHCB1.2* as a prototype PhANG showed an even greater decrease in transcript level in the double than in *cue8* (something associated with the much more pronounced plastid defect, but surprising for plants carrying the *gun1* mutation), while the PhANG-driving GLKs were as reduced (figure 7*d*). We conclude that either GUN1 plays no role in the retrograde repression of PhANGs in *cue8*, or that the *cue8* mutation itself impairs the action of GUN1.

**Figure 8.**
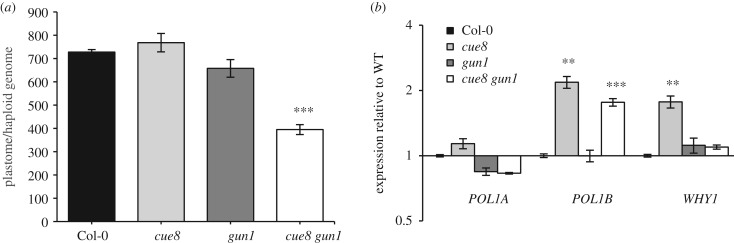
Maintenance of the number of copies of the plastome necessitates GUN1 activity. (*a*) Number of copies of the plastid genome in WT and the mutants indicated, expressed per copy of the haploid nuclear genome. (*b*) Transcript levels of the two organellar DNA polymerases in seedlings of each genotype, and of transcripts for the nucleoid-associated WHIRLY1 protein, quantified and expressed as in figure 3*a*.

The fact that expression of NEPs is elevated in the double mutants (figure 7*c*), but the expression of NEP-driven transcripts in their chloroplasts is not (figure 7*b*), also raises an apparent paradox. The paradox is resolved by the fact that *cue8 gun1* double mutants fail to sustain cpDNA replication, as demonstrated by their levels of cpDNA copies per haploid genome being only half those of single mutants or the WT (figure 8*a*). In the double, expression of *POL1B* was still elevated, but that of *WHY1*, which encodes an abundant nucleoid protein [29,30], was not (figure 8*b*). In summary, it appears some aspects of the chloroplast ‘corrective’ response (elevated transcripts of the NEPs and of the organellar DNA POL, in addition to the decrease of those of PhANGs) are maintained even in the absence of GUN1, but others (elevated NEP activity, maintained nucleoids in the developmentally reduced plastids) are not, and this impaired corrective response coincides with complete failure of chloroplast development and eventual seedling lethality.

### (g) RNA editing in plastids is altered by the loss of CUE8, even in the absence of GUN1

**Figure 9.**
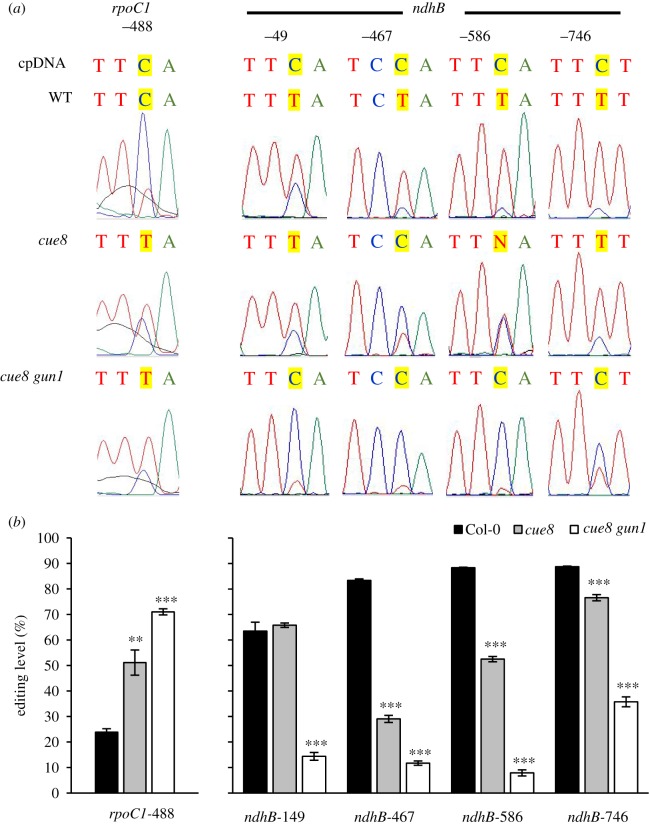
Loss of CUE8 triggers changes in editing of two plastid mRNAs, and this does not require GUN1 activity, the changes being exacerbated in the double mutant. (*a*) Representative sequence electropherograms of cDNA generated from WT, *cue8* and *cue8 gun1* cDNA. The original, genomic cpDNA sequence is indicated at the top. (*b*) Quantitation of the degree of editing of the two plastid mRNAs shown in (*a*). The average of three independent cDNA preparations from different seedling samples per genotype is shown. Error bars represent s.e.m. between biological replicates, and asterisks significance of difference against the WT Col-0. (Online version in colour.)

mRNA editing makes specific C-to-U base substitutions in a number of chloroplast transcripts, and it has been reported that plastid defects triggered by the loss of TOC159 (in the *ppi2* mutant), by bleaching herbicide or by plastid translation inhibition, all trigger consistent defects in editing [16]. Notably, although the observed editing defects in *ppi2* did not require GUN1 [16], a wide survey of editing efficiencies has revealed many to be altered under plastid-defective conditions in the *gun1* mutant, with GUN1 being directly involved by interaction with the MORF2 editing factor [17]. We asked whether mRNA editing efficiency had been altered by the loss of CUE8 at two selected mRNAs: *rpoC1*, previously shown to have increased incidence of editing in plastid-defective conditions, and *ndhB*, showing the opposite response [17]. Indeed, the loss of CUE8 led to a higher frequency of editing in *rpoC1* and lower at three of the four sites of *ndhB* (figure 9), in line with the effects of other plastid defects [17]. GUN1 was not required for these changes; in fact, the efficiency of editing further increased for *rpoC1* and further decreased for *ndhB* transcripts in the *cue8 gun1* double mutant (figure 9). Thus, we confirm that changes in plastid mRNA editing occur in *cue8*, but surprisingly we observe no role for GUN1 in such changes.

## 4. Discussion

**Figure 10.**
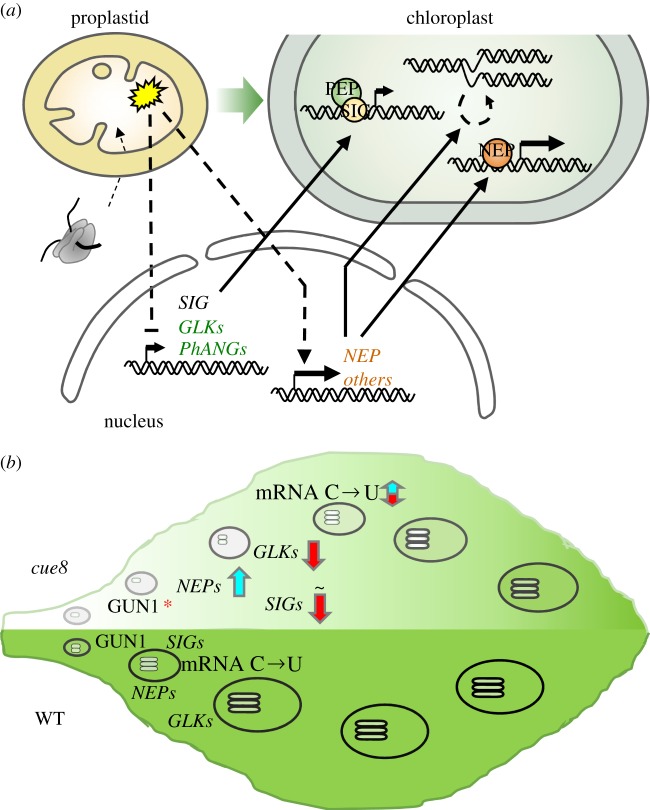
Model summarizing the retro-anterograde, compensatory response to early chloroplast biogenesis failure, and how that response represents maintenance of an early plastid development state, underlays the phenotype of *cue8* and possibly also that of other virescent mutants or conditions. (*a*) Early chloroplast biogenesis failure triggers one or more retrograde (chloroplast-to-nucleus) signalling events (generation of a negative signal or loss of a positive signal) which results in reduced expression of PhANGs and their regulators and of specific sigma factor genes in the nucleus, while also promoting the expression of NEPs and actively maintaining the replication state of the cpDNA; together these processes amount to an anterograde (nucleus-to-chloroplast) compensatory response to the original *cue8*-triggered retrograde signal. (*b*) Activity of GUN1 at early stages of chloroplast biogenesis senses the impaired organellar development in *cue8*, triggers a retrograde signalling event, and this leads to an anterograde corrective response which essentially extends the very early expression state of the plastid-targeted proteins indicated, and probably others (note that the same processes occur very early in the WT, at leaf primordia stages much younger than that visualized here). This prolonged ‘early organelle development’ state in *cue8* may lead to slow but successful chloroplast biogenesis in photosynthetic cells, manifested as virescence. The adaptive significance of this retro-anterograde check on chloroplast biogenesis is demonstrated by the loss of virescence and catastrophic chloroplast developmental failure in *cue8 gun1* double mutants. (Online version in colour.)

The nuclear gene expression defects of *cue8* were previously found to be specific to PhANGs [12,23]. At face value one would have expected cpDNA-encoded genes to also be equally broadly reduced in expression. This, to our initial surprise, was not the case; we saw a reduced expression of PEP-transcribed genes, many of which encode photosynthetic proteins, and elevated expression of NEP-transcribed genes, generally encoding plastid housekeeping proteins including subunits of the PEP itself. However, such a contrasting phenotype had first been observed upon the deletion of a PEP subunit in transplastomic tobacco [24] and has subsequently been observed associated with defects of the plastid genetic machinery. Indeed, the PEP associates with DNA in nucleoids which can be isolated as ‘transcriptionally active chromosomes' (pTACs), containing a number of cofactors referred to as pTAC proteins. Defects in several pTACs lead to a common phenotype, including this organellar gene expression response and albinism [31]. The decrease in PEP-driven and relative elevation of NEP-driven transcripts is very prominent in seedlings defective in MRL7/RCB [25], recently shown necessary for assembly of the PEP [26], or lacking ANU7, a DnaJ-like chloroplast protein of unknown molecular function [32]. In those cases, the response was presumed to be compensatory, an attempt to correct the defective plastid genetic function. Elements of the mechanism of correction emerged later from global gene expression analyses [32,33]. In addition to those observations, other elements are reported here for the first time. Furthermore, we provide evidence for both the corrective value of such a response and for its adaptive significance, as deduced from the consequences of its loss. Overall, our data are consistent with a mechanism for this correction which involves a two-step process summarized in figure 10*a*: plastid-to-nucleus retrograde signalling of the organellar defect results in changes in nuclear gene expression for proteins that are responsible for anterograde control of plastid gene expression. On the one hand, the expression of genes for sigma factors, determining PEP DNA-binding and promoter specificity, is reduced. On the other, the expression of the *RPOT* genes encoding plastid NEP itself is elevated. This is accompanied by a third element of the response, an observed ability to fully maintain the number of cpDNA copies per cell, in spite of the delayed chloroplast development. While we confirm cpDNA replication occurs very early in plastid development, a further increase is associated with cellular and chloroplast differentiation, and even that further increase had occurred in *cue8* in spite of the delayed chloroplast differentiation. Each of these three elements of anterograde correction is necessary for the complete response.

Inhibition of PEP-mediated chloroplast transcription with rifampicin causes a decreased accumulation of PhANGs, and it has been reported that SIG2 and SIG6 are the two sigma factors whose function is particularly important for PhANG maintenance [33]. We, however, observed at different stages or in different genotypes reductions in SIG1, SIG2, SIG4 and SIG5 expression. Hence the sigma factors whose defect triggers ‘retrograde signalling’ do not fully overlap with those altered as part of the plastid response. In fact, consistent with our observations, Woodson *et al*. [33] observed that arrest of chloroplast differentiation with norflurazon reduces the transcript accumulation of SIG1 and SIG2, as well as SIG4. SIG1 has been shown to play a relatively global role, SIG2 helps transcribe tRNAs but these include the photosynthetically quantitatively important *trnE*, the precursor of tetrapyrrole pigments, and SIG5 recognizes environment- or stress-responsive promoters [34]. The roles of sigma factors in *Arabidopsis* involve a combination of specificity and overlap [35], and indeed the functions have evolved and differ somewhat between dicots and monocots [36].

The elevated expression of NEP-encoding *RPOT* genes which we observed is consistent with the increased expression seen through global expression analysis of the chloroplast and leaf development-defective *anu7* mutant [32]. Can elevated NEP expression be at least in part responsible for a ‘compensatory’ response? Current evidence would argue for this to be the case. Complete arrest of PEP activity led initially to a view of a complete, qualitative, binary distinction between NEP-driven (early, housekeeping) and PEP-driven (later, photosynthetic) transcription [24]. Such a distinction is overly simplistic, since NEP and PEP are both active early in seedling development in *Arabidopsis* [37], and several transcripts are polycistronic and encode proteins of both genetic and photosynthetic function [34]. Global plastid transcript analysis in a developing barley leaf to identify promoter use, and consequently polymerase origin [38], has also observed the activity of both NEP and PEP throughout leaf development. Nevertheless, clear quantitative differences were also evident, with NEP activity being particularly important at the early stages of plastid development. Such a quantitative, preferential functional role becomes particularly strongly supported by the phenotypes of mutants: the *ΔrpoB* mutant of tobacco [24] and mutants from the loss of different pTACs in *Arabidopsis* [31] are albino but heterotrophically viable. By contrast, simultaneous loss of the two *RPOT* genes (while retaining the gene for the mitochondrial-targeted enzyme) results in very early growth arrest [39]. This differential role of the two plastid transcription systems is necessary to understand the selection pressure for the evolution of their differential regulation which we observe in *cue8*.

The role for the cpDNA copy number maintenance in the virescence-associated compensation has, to our knowledge, not been observed previously. The number of copies of the plastid genome is limiting for the accumulation of transcripts overall [40], although the response of different transcripts varies. cpDNA replication occurs early in development [41,42]. Although it is complete in cotyledons of 5-day-old *Arabidopsis* seedlings [27], and although we observed that as a proportion of nuclear DNA, it reaches a stable value soon after germination, we also saw that a further increase (around an extra third) occurred during seedling establishment as the nuclear ploidy increased. It is therefore still remarkable that the low plastid occupancy in young *cue8* cotyledon cells, with cells differentiating well ahead of chloroplasts, is not accompanied by reduced cpDNA amounts. In other words, the dense accumulation of cpDNA reflects a juvenile plastid stage accompanied by a small further boost in replication. Organellar DNA replication uses a polymerase shared between mitochondria and chloroplasts, encoded in *Arabidopsis* by two paralogous genes, loss of one of which, *POL1B*, can be tolerated but results in plants particularly sensitive to low doses of a drug causing double-strand breaks [43]. Remarkably, such treatment leads specifically to virescent *pol1B* plants. Consistently, of the two, *POL1B* is more abundantly expressed in meristematic regions [44]. This is the isoform whose expression was elevated in *cue8*. We cannot conclude that this elevation is responsible for the maintenance of cpDNA in *cue8*, and the regulators of cpDNA replication are poorly understood [45]. WHIRLY1 is a major component of pTAC, and so can serve as a marker of nucleoid assembly [30], but is unlikely to be a positive regulator of cpDNA replication since its loss actually results in diffuse nucleoids which contain more, not fewer, copies of cpDNA [29]. We can, however, confirm that such maintenance of cpDNA is essential for the elevated expression of NEP-transcribed genes, since *cue8 gun1* exhibited elevated nuclear *RPOT* expression, but failed to accumulate sufficient cpDNA copies and exhibited no elevation of plastidic NEP-driven transcripts.

A fascinating aspect of the response is the alteration of plastid transcripts' editing. Organellar mRNA editing alters the sequence of the encoded polypeptides, and therefore their function [46]. Inhibition of carotenoid synthesis with norflurazon, or of plastid translation with lincomycin, or loss of a plastid protein import component all lead to consistent decreases in editing efficiency at a number of sites, particularly of the *ndhB* transcripts [16]. However, the editing efficiency in *rpoC1* transcripts actually increases under norflurazon or lincomycin [17]. It is remarkable that we observed the same, contrasting changes in editing efficiency in *cue8* (reduced for *ndhB*, increased for *rpoC1*), and that this parallels the increased expression of *rpoC1*. The biological significance of mRNA editing and its changes is very poorly understood. A wider analysis would be necessary to confirm this, but our limited observations raise the intriguing possibility that expression and editing changes mirror each other, or even that they share a biological role.

In summary, an early plastid biogenesis defect has triggered a retrograde biogenic signal which has altered ‘organelle developmental’ nuclear gene expression. This has altered the response of important nuclear genes, both photosynthetic––*LHCB1*, others [23]––and regulatory––*GLK1* and *GLK2* [47]. Part of the response has led to corrective action in the plastids, both to suppress photosynthesis-associated, preferentially PEP-transcribed genes and to compensate for housekeeping functions, through elevated NEP expression and maintained genome copy number. Concomitant changes in chloroplast RNA editing have taken place. In this regard, ‘biogenic’ and ‘operational’ chloroplast signals share scope. For example, stress-induced operational chloroplast signals trigger the expression of nuclear stress-protectant genes [48], but also have an ‘anterograde’ impact altering chloroplast genetic function [49].

Which is the source and signal of the retro-anterograde response documented here? We attempted to answer this question through the examination of the response of seedlings with chloroplasts with contrasting defects [2]. The result was not absolutely conclusive: a more consistent outcome of elevated NEP and NEP-driven mRNA levels was observed in the seedlings with deficiency primarily in the import of housekeeping proteins, but it was apparent in both genotypes tested. One can conclude that defects in the import of proteins required early in chloroplast biogenesis (regardless of the import receptor) can trigger the response. GUN1 [5] has emerged as a central integrator of alterations in chloroplast development which cause defective tetrapyrrole metabolism [5,13], protein translation [13,50] and protein import [13,15,51], the latter leading to an unfolded protein stress response in the cytosol [15]. Indeed GUN1 clearly plays a role in the retro-anterograde compensatory response (figure 8). However, the action of GUN1 should not be overestimated; we found that elevated NEP expression relative to WT still occurred in *cue8* in the absence of GUN1, while the maintenance of cpDNA copies to WT levels failed to occur. Remarkably, the removal of GUN1 failed to trigger a ‘genomes uncoupled’ phenotype in *cue8* (figure 8). Simultaneous loss of GUN1 and PPI2 (TOC159) also leads to eventual seedling lethality of the *ppi2* mutant but, in contrast with our case, an attenuated PhANG expression reduction (a ‘genomes uncoupled’ phenotype) is still seen [16]. In other words, either GUN1 is not responsible for the retrograde suppression of PhANG expression in *cue8*, or loss of CUE8 has also impaired this function of GUN1. A ‘genomes uncoupled’ phenotype is seen in *gun1* as an elevation of PhANG expression relative to WT when plastid development is inhibited, but such expression is still much reduced relative to plants with functional plastids [5,28], i.e. it would be appropriate to describe the mutant as ‘genomes *partially* uncoupled’. It has recently been shown that retrograde signalling involves a major control of protein translation and stability, and such control, particularly for ribosomal plastid proteins, is fully present, indeed even stronger, in *gun1* [52]. Similarly, we observed that the changes (both positive and negative) in chloroplast mRNA editing which occur in *cue8* are enhanced in *cue8 gun1*, further evidence for an impaired GUN1 function in *cue8*.

Consideration of the available data reveals some interesting similarities between the ‘retro-anterograde compensation’ mechanism we describe here and the stages of chloroplast biogenesis during leaf initiation. Plastid division has been shown to occur early during monocot leaf development, prior to greening [53], and according to our observations, division is certainly not impaired in *cue8*; it could even be increased. Very early stages of chloroplast development involve greater levels of activity in chloroplasts of the NEP, while the PEP reaches maximum activity in green tissues [38,40]. Those early stages also involve rapid replication of cpDNA [41]. Very early leaf development is also the time at which accumulation of GUN1 can be exclusively detected [54]. In other words, the response observed in *cue8* and probably in other mutants with fundamental defects in plastid development, resembles a ‘juvenile’ phase of chloroplast biogenesis (figure 10*b*). This view is satisfyingly consistent with the virescent phenotype (figures 1 and 2). If this line of reasoning is extended one could argue that, rather than mutants with defective plastids showing ‘repression’ of PhANG expression, they never reach the phase in which the activation of such genes occurs.

Such ‘delay and allow time to correct’ response could explain the widespread nature of virescent phenotypes, caused by defects in metabolism [9], in protein homeostasis [7,8], or even in genome stability [43]. It does not suffice to enable successful chloroplast biogenesis under some conditions (e.g. growth on norflurazon), but it does in others (e.g. the mutations here discussed). While GUN1 may not be entirely responsible for the response, the fact that its loss converts a virescent phenotype into a lethal one also highlights the adaptive significance of retrograde communication (and of its anterograde follow-up). Loss of TOC159, a component of the plastid protein outer translocon, or of PRPL11, a plastid ribosomal protein, have been also observed to result in synthetic lethality when combined with the loss of GUN1 [13,55]. Such observations demonstrate not just the fascinating, intriguing nature of the pathways of interorganellar communication, but also their fitness value.

## Supplementary Material

Supplementary tables and figures

## Data accessibility

This article has no additional data.
