# Supplementary material for: Retrograde signalling in a virescent mutant triggers an anterograde delay of chloroplast biogenesis that requires GUN1 and is essential for survival
Source: Philos Trans R Soc Lond B Biol Sci. 2020 May 4;375(1801):20190400. doi: 10.1098/rstb.2019.0400 (PMC7209947; doi:10.1098/rstb.2019.0400)
Supplement: Supplementary tables and figures [file rstb20190400supp1.pdf]

Loudya et al. 2020, Retrograde signalling in a virescent mutant.
Figure S1

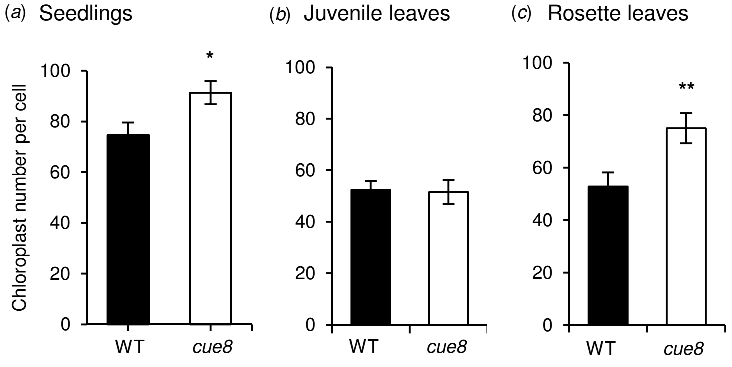

Average chloroplast number in cells of *cue8* and its wild type (WT) at the stages indicated: (a) Seedlings equivalent to those in Figure 2a and 2d. (b) Young leaves equivalent to those in Figure 2b and 2e. (c) Mature leaves equivalent to those in Figure 2c and 2f. Average number per cell. Error bars represent s.e.m. (n=10-13).

Loudya et al. 2020, Retrograde signalling in a virescent mutant.
Figure S2

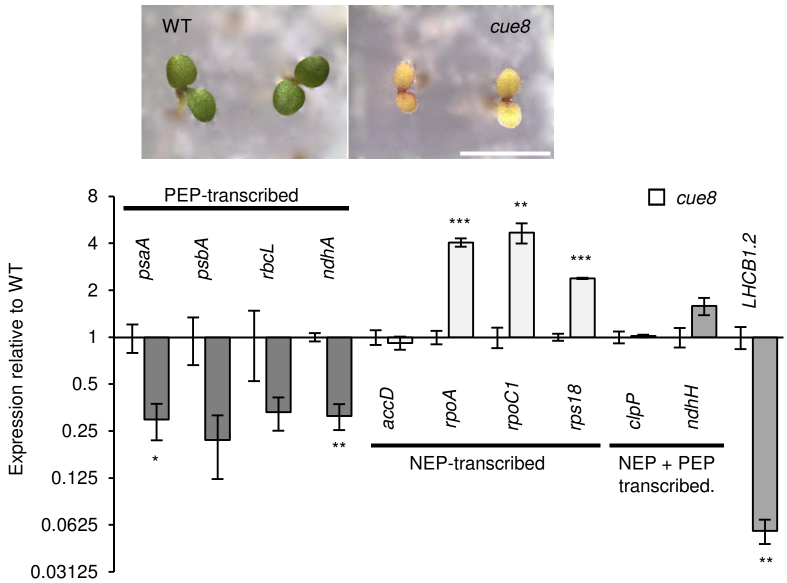

Plastid-encoded gene expression phenotype of 5 day old *cue8* and WT seedlings. (a) 5 day old seedlings of WT and *cue8* (image of WT reproduced from Figure 3). Scale bar 5 mm. (b) Plastid-encoded gene expression phenotype of 5 day old *cue8* seedlings relative to 5 day old WT seedlings, mutant seedlings being time-matched but developmentally delayed. Levels of plastid genome-encoded transcripts were quantified and expressed as in Figure 3a.

Loudya et al. 2020, Retrograde signalling in a virescent mutant.
Figure S3

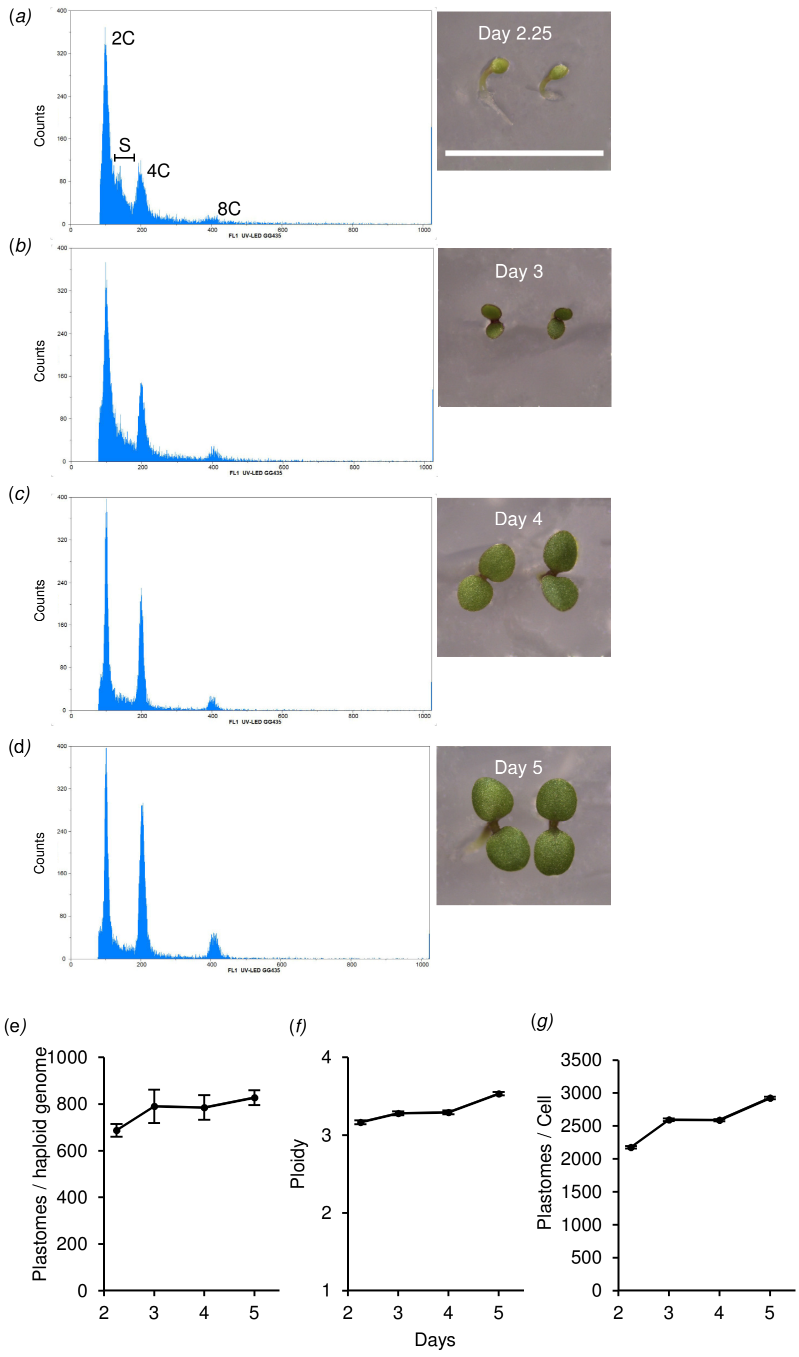

Ploidy analysis of WT developing seedlings. (a-d) Ploidy analysis of WT 2.25 to 5 day old seedlings, and images of corresponding, example seedlings. Scale bar: 5 mm. C represents the haploid DNA content, and S the DNA synthesis phase. (e) Number of copies of the plastid genome in WT at the different ages, expressed per copy of the haploid nuclear genome. Error bars represent s.e.m. (3 samples per time point). (f) Average cellular ploidy levels, calculated from the proportion of nuclei at 2C, S, 4C or 8C stages (6 to 7 samples per time point). (g) Number of cpDNA copies per cell, calculated using values in (e) and (f).

Loudya et al. 2020, Retrograde signalling in a virescent mutant.
Figure S4

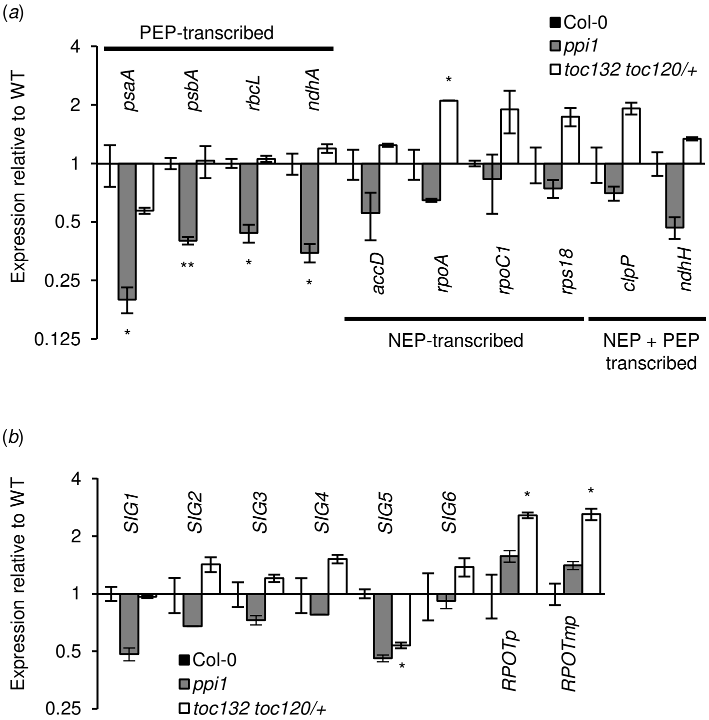

Gene expression phenotype of juvenile (<4mm long) leaves of mutants impaired primarily in photosynthesis or housekeeping chloroplast functions, as a result of selective protein import defects, share the cue8 chloroplast gene expression phenotype, although loss of housekeeping chloroplast function does so more consistently. (a) Levels of plastid genome-encoded transcripts in juvenile leaves of Col-0 WT and mutant genotypes, quantified by qRT-PCR, normalised and displayed as in Figure 3a. (b) Transcript levels in WT and mutant juvenile leaves of nuclear-encoded genes for PEP specificity-determining sigma factors (*SIG*) and for NEPs (*RPOTp* and *RPOTmp*), expressed and displayed as in Figure 3c.

Loudya et al. 2020, Retrograde signalling in a virescent mutant. Electronic suppl. material

Table S1

Primers used for genotyping

**Mutant**	**Border/ forward / reverse**	**Sequence**	**Enzyme digestion**	**Band size**
toc120	FISH1-2	CTGGGAATGGCGAAATCAAGG	-	-
	toc120-2_F	CAAAAGTGGAGCAGCACGAC		
	toc120-2_R	AACCAGGTTAGATTCTCGCCA		
gun1-1	gun1-dCaps-F	TAACTATTGCTAAGAGGATTTTCGAAACAG	*AluI* (*GUN1*)	*gun1* (99bp) *GUN1* (69bp)
	gun1-dCaps-R	CACTTCTCCCATAAGCGCTGA		

Table S2

Primers used for gene expression analysis of nucleus-encoded genes

Gene	Locus	Forward / Reverse	Primer sequence
UBQ10	AT4G05320	UBQ10-F	GGAGGATGGTCGTACTTTGG
		UBQ10-R	TCCACTTCAAGGGTGATGGT
LHCB1.2	AT1G29910	LHCB1-F	CCGATCCAGTCAACAACAAC
		LHCB1-R	TCAAACCATCACATACAACCTTC
RPOTp	AT2G24120	RPOTp -F	CTTGGTGATTGTGCAAAGATAATT
		RPOTp -R	GGGAGGAAATGCAGTTCTTTGTT
RPOTmp	AT5G15700	RPOTmp-F	CATATGATGATGACTGCGGTTG
		RPOTmp-R	TATCCACATCACACGCATGC
SIG1	AT1G64860	SIG1-F	AACTAAAACACGCAGCGAGGA
		SIG1-R	TCTTAAGGATCATTGCCTCCATTT
SIG2	AT1G08540	SIG2-F	TTGGACAAAGTGTTGGACTCGT
		SIG2-R	CTTCATTCTCCCATCCTCCATC
SIG3	AT3553920	SIG3-F	CCCACATACCCTGCCTGAA
		SIG3-R	TGTAGAGATGAACTGGTGAAAAGCA
SIG4	AT5G13730	SIG4-F	CGGGGACCAGATGAAACAA
		SIG4-R	GCCCAAAGTAAAGTCCCAATACAC
SIG5	AT5G24120	SIG5-F	TGGAGCTAATAACAGCAGACAGC
		SIG5-R	TCGGCTTCAATGAATCGAGCAC
SIG6	AT2G36990	SIG6-F	CTTATTGCAGGAAGGAAGCATGGG
		SIG6-R	TCTGCATCCGGATTGCGGTTTG
GLK1	AT2G20570	GLK1_F	TTGGGTCTCCGATTCTCCCTAT
		GLK1_R	GCAACTGGCGGTGCTCTAAAT
GLK2	AT5G44190	GLK2_F	ACCGTACTGGCATCAGCAAC
		GLK2_R	TGAATGTCGATGGGAGGATT
POL IA	AT1G50840	POL IA-F	TTCCGGCGTCAAAGTCACGTGC
		POL IA-R	TGCACTTCCCTGGACTGGAGTGT
POL IB	AT3G20540	POL IB-F	CCTGAATACCGTTCACGTGCCCA
		POL IB-R	AGCCGCACTTCCCTGAACAGGA

WHY1	AT1G14410	WHY-F	CTGGTGCTCTTGGGTCCACTGT
		WHY-R	TTCGAGAAGCAGAGGTTCG

Table S3

Primers used for gene expression analysis of chloroplast-encoded genes

Gene	Locus	Forward / Reverse	Primer sequence
psaA	ATCG00350	psaA-F	GCCAAGAAATCCTGAATGGA
		psaA-R	CATCTTGGAACCAAGCCAAT
psbA	ATCG00020	psbA-F	GAGCAGCAATGAATGCGATA
		psbA-R	CCTATGGGGTCGCTTCTGTA
rbcL	ATCG00490	rbcL-F	AGGAACTTTAGGCCACCCTTGG
		rbcL-R	TGCTTCCAGAGCTACTCGGTTG
ndhA	ATCG01100	ndhA-F	GGATGGAATTTGTGGCGTCAACC
		ndhA-R	ACGGTAACCTCTCGCATTCTGC
clpP	ATCG00670	clpP-F	GTCGGAGGAGCAATTACCAA
		clpP-R	GTGATGGTTTCGCGAAGTTT
ndhH	ATCG01110	ndhH-F	ATGGGAAATTCAATGGCAAA
		ndhH-R	TCAAAGCCCCTGCTTTCTAA
accD	ATCG00500	accD-F	TGTGGATTCAATGCGACAAT
		accD-R	TTTTGCGCAGAGTCAATACG
rpoA	ATCG00740	rpoA-F	GCGATGCGAAGAGCTTTACT
		rpoA-R	CCAGGACCTTGGACACAAAT
rpoC1	ATCG00180	rpoC1-F	CTCGGTGATTGTCGTTGGACCTTC
		rpoC1-R	ATTTCGCGAGGCAATCCACAGC
rpS18	ATCG00650	rpS18-F	CAAGCGATCTTTTCGTAGGC
		rpS18-R	AAAGTCACTCTATTCACCCGTCT

Table S4

Primers used for quantitation of chloroplast genome copy number

Gene	Locus	Forward / Reverse	Primer sequence
HO1	AT2G26670	AtHO1-F	CCCCAACTCTCAAGATTCCA
		AtHO1-R	CCGCAACCACCACTAAAGAC
CHS	AT5G13930	AtCHS-F	TGAGATCAGACAGGCTCAGAGA
		AtCHS-R	ACTGTTGGTGATGCGGAAGT
rbcL	ATCG00490	AtrbcL-F	TTCGGTGGAGGAACTTTAGG
		AtrbcL-R	GCAAGATCACGTCCCTCATT
ndhG	ATCG01080	AtndhG-F	GGGAATGGGATTACTTCGTTGG
		AtndhG-R	ACCCCGTACCATGACGTATC
ycf2	ATCG00860	AtYcf2-F	TGGAAAAGGCCCGTCTCAAT
		AtYcf2-R	CCACCGCACGAAGAAAATGT

Table S5

Primers used in the analysis of RNA editing for PCR and sequencing

Gene/ editing site	Locus	Forward / Reverse	Sequence	Purpose
rpoC1- 488	ATCG00180	rpoC1-488F	TTTTCTTTTGCTAGGCCCATAA	PCR
		rpoC1-488R	TTCGCAAATCTAAATCGGCT	PCR
		rpoC1-593SR	GCACCCGCCCCAGTAGAA	Sequencing
ndhB- 149, *ndhB*- 467, *ndhB*- 586, *ndhB*- 746	ATCG00890	ndhB-1F	GCCTTTCATTTGCTTCTCTT	PCR
		ndhB-1R	TCCTTCGTATACGTCAGGA	PCR
		ndhB-346SR	CGGATAGAGGAATACAGAGAGTTGA	Sequencing
		ndhB-271SF	TTCCAAACGAACAATTTCAACG	Sequencing
